# CANT-HYD: A Curated Database of Phylogeny-Derived Hidden Markov Models for Annotation of Marker Genes Involved in Hydrocarbon Degradation

**DOI:** 10.3389/fmicb.2021.764058

**Published:** 2022-01-07

**Authors:** Varada Khot, Jackie Zorz, Daniel A. Gittins, Anirban Chakraborty, Emma Bell, María A. Bautista, Alexandre J. Paquette, Alyse K. Hawley, Breda Novotnik, Casey R. J. Hubert, Marc Strous, Srijak Bhatnagar

**Affiliations:** ^1^Energy Bioengineering and Geomicrobiology Group, Department of Geoscience, University of Calgary, Calgary, AB, Canada; ^2^Energy Bioengineering and Geomicrobiology Group, Department of Biological Sciences, University of Calgary, Calgary, AB, Canada

**Keywords:** hydrocarbon degradation, Marker genes, Hidden Markov Models, gene annotation, hydrocarbon cycling

## Abstract

Many pathways for hydrocarbon degradation have been discovered, yet there are no dedicated tools to identify and predict the hydrocarbon degradation potential of microbial genomes and metagenomes. Here we present the Calgary approach to ANnoTating HYDrocarbon degradation genes (CANT-HYD), a database of 37 HMMs of marker genes involved in anaerobic and aerobic degradation pathways of aliphatic and aromatic hydrocarbons. Using this database, we identify understudied or overlooked hydrocarbon degradation potential in many phyla. We also demonstrate its application in analyzing high-throughput sequence data by predicting hydrocarbon utilization in large metagenomic datasets from diverse environments. CANT-HYD is available at https://github.com/dgittins/CANT-HYD-HydrocarbonBiodegradation.

## Introduction

Hydrocarbons are diverse compounds consisting of carbon and hydrogen atoms that differ in size, structure, and reactivity. They can be the product of geological processes as well as produced biogenically by organisms in all domains of life (Tornabene et al., 1969; Lerdau et al., 1997; Lea-Smith et al., 2015). Assessing hydrocarbon use by microorganisms, as a source of carbon and/or energy, is important for evaluating the consequences of hydrocarbon presence or contamination (Atlas and Hazen, 2011), understanding the global carbon cycle (González-Gaya et al., 2019), and for industrial applications, such as the synthesis of biocatalysts (Prier and Kosjek, 2019). Degradation of hydrocarbon molecules is kinetically challenging due to the chemical inertness of the organic C–H bond, and when present, the stability of aromatic ring structures (Rabus et al., 2016). Microorganisms employ a range of enzymes to use hydrocarbons (Rabus et al., 2016; Xu et al., 2018) in oxic and anoxic conditions. Catabolism of these hydrocarbons is coupled with reduction of terminal electron acceptors such as oxygen, nitrate, sulfate, and iron or *via* syntrophy with methanogens (Zhang et al., 2019).

The discovery of hydrocarbon degrading microorganisms has traditionally relied on cultivation in the laboratory using hydrocarbon substrates (Rueter et al., 1994; Kniemeyer et al., 2007). Successful cultivation preceded the identification of genes involved in hydrocarbon metabolism with techniques such as gene knockouts, protein expression analyses, and gene sequencing (Schneiker et al., 2006; Wang and Shao, 2014; Gregson et al., 2018; Wang et al., 2018; Liu et al., 2019; Li et al., 2020). These studies are crucial for providing fundamental knowledge on the ever-growing diversity of hydrocarbon degrading microorganisms as well as uncovering new degradation pathways. The recent exponential rise in sequence data and the consequential increase in known microbial diversity have provided new opportunities to explore hydrocarbon degradation potential in diverse environments and uncultured microorganisms. One approach for exploring sequence data is to annotate genes using Hidden Markov Model (HMM). HMMs are trained on the multiple sequence alignments of amino acid sequences and produce position-specific scores and penalties when searching query sequences. HMMs have better sensitivity and recall for identifying homologs of conserved protein domains, compared to conventional pairwise alignment tools such as blastp (McGinnis and Madden, 2004), which use a position-independent scoring matrix (Eddy, 2004). Detection of metabolic potential in whole genomes or metagenomic datasets is generally accomplished using functional annotation tools aided by HMM databases such as KEGG (Kanehisa and Goto, 2000) and Pfam (Finn et al., 2014). While these large databases can confidently identify central metabolic and other well studied pathways, specific HMMs and tools for accurate annotation of catalytic genes in hydrocarbon degradation pathways are currently lacking. Genes involved in hydrocarbon degradation can share sequence similarity to genes from other metabolic pathways and consequently, are often misannotated (Callaghan et al., 2008; Khelifi et al., 2014). Hence, there is a need for a purpose-built tool for the accurate detection of hydrocarbon degradation pathways in sequence data.

Here we present the Calgary approach to ANnoTating HYDrocarbon degradation genes (CANT-HYD), a database of 37 HMMs designed for the identification and annotation of marker genes that are critical for the aerobic and anaerobic degradation of alkane and aromatic hydrocarbons. CANT-HYD is tested and validated against 72 genomes of known hydrocarbon degrading bacteria, representing a broad spectrum of hydrocarbon metabolism. Using these validated HMMs, over 30,000 representative genomes covering the entire bacterial and archaeal tree of life are analyzed to identify hydrocarbon degrading microorganisms. Forty-one publicly available metagenomes from diverse environments are also analyzed using CANT-HYD to explore hydrocarbon degradation potential in diverse environments. Lastly, we compare the performance of CANT-HYD HMMs to their counterparts from eggNOG, Pfam, and KEGG Orthology (KO) databases.

## Methods

### Selection and Clustering of Archetype Reference Sequences

**FIGURE 1:**
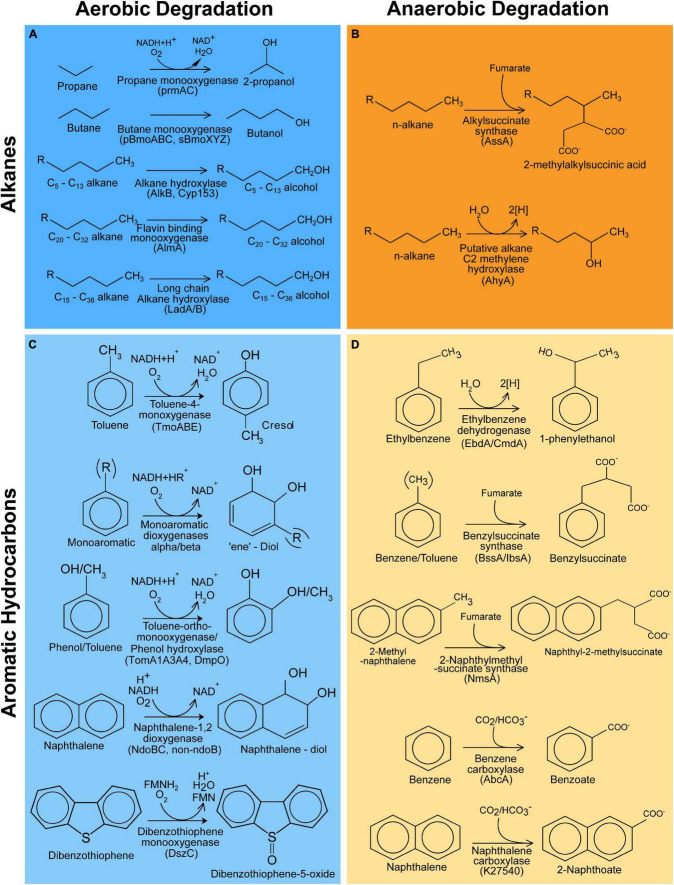
Hydrocarbon degradation reactions covered by CANT-HYD. Reactions for the degradation of alkanes through aerobic **(A)** and anaerobic pathways **(B)** and degradation of aromatic hydrocarbons through aerobic **(C)** and anaerobic **(D)** pathways.

Enzymes involved in the activation of hydrocarbon substrates in aerobic and anaerobic hydrocarbon degradation pathways of aliphatic and aromatic compounds were identified through a literature search (Figure 1). Amino acid sequences encoding the catalytic subunits of these enzymes were obtained from Genbank and were classified as either “experimentally verified” or “putative.” The “experimentally verified” sequences refer to amino acid sequences from published studies with experimental proof of the intended function. Experimental proof consisted of gene cloning or protein purification and corresponding enzyme assays, or gene knockout studies. Gene sequences labeled “putative” refer to sequences with strong evidence of function but lacking these definitive prerequisite analyses. Putative sequences often originated from isolates or enrichment cultures where there is evidence of hydrocarbon degradation or genomic and/or proteomic evidence for the enzyme responsible. The resulting curated 105 amino acid sequences from 53 different species (Supplementary Table 1) are referred henceforth as “archetype” as they represent the gene sequence pattern for a verifiable hydrocarbon degradation function. Amino acid sequences were clustered into homologous groups based on ≥20% amino acid identity as determined by blastp v2.9.0 (McGinnis and Madden, 2004) to place related archetype sequences on the same phylogenetic tree and reduce the downstream computational resource requirements. A loose grouping as carried out here is unlikely to affect the final HMM, as manual curation and pruning of phylogenetic trees in downstream processing took this clustering into account. Archetype sequences that did not cluster at 20% were either manually added into homologous groups of similar function or left as singletons.

### Homology Search to Obtain Sequences Similar to Archetypes

Amino acid sequences sharing sequence homology to the 105 archetype genes were recruited from the NCBI non-redundant (nr) protein database using a Diamond homology search (Buchfink et al., 2014). All hits with query coverage ≥70% and e-value ≤10^–4^ were retained as putatively phylogenetically related sequences and added to the query’s homologous group. The sequences of homologous groups were dereplicated, followed by clustering at 98% amino acid identity using the USEARCH v9.0.2 *derep_fulllength* and *cluster_fast* commands (Edgar, 2010). The resulting sequences from the Diamond homology search are referred to as the “DIAMOND sequences” database and were used in downstream steps of HMM construction, and to generate cutoff scores for the HMMs.

### Grouping Genes With Similar Functions Using Phylogenetic Analysis

A multiple sequence alignment was generated for each clustered (98%) homologous group using MUSCLE v3.8.31 (Edgar, 2004). The alignments were used to create maximum-likelihood trees using FastTreeMP v2.1 (Price et al., 2010) with the parameters –*pseudo* and *-spr 4*. Trees were manually inspected using iTOL v5.6.3 (Letunic and Bork, 2019) or Dendroscope v3.6.3 (Huson et al., 2007) to identify monophyletic clades of genes containing experimentally verified archetype sequences (Supplementary Figure 1). These monophyletic clades were easily identifiable as archetype clustering was carried out at a low threshold (on ≥20% amino acid identity) and thus a substrate-specific set of seed sequences was extracted from each clade. In some instances, where a clear monophyletic distinction was lacking, a broader function-specific set of seed sequences were extracted (e.g., MAH_alpha group includes TcbA, IpbA, BnzA, and BphA).

### Processing Homologous Groups With >5,000 Sequences

**FIGURE 2:**
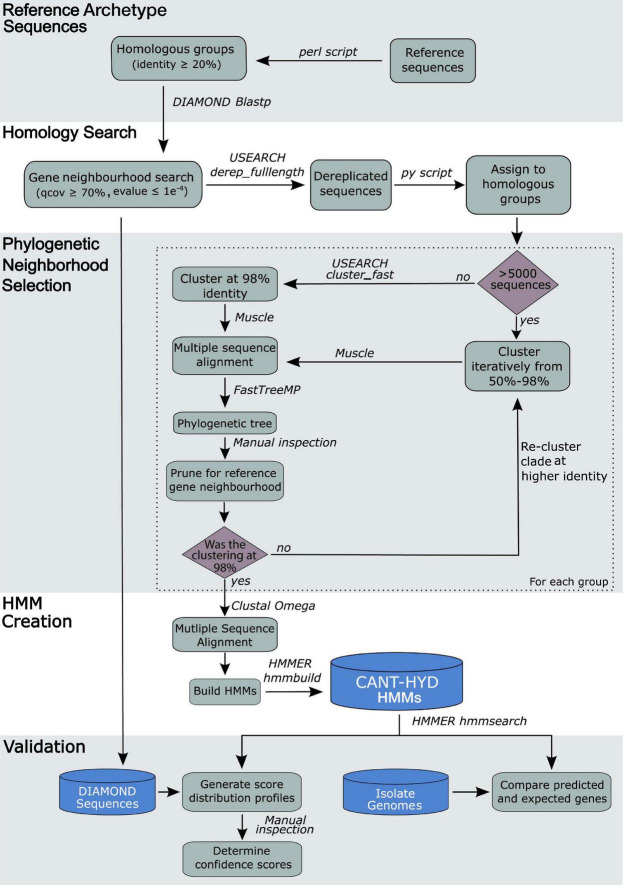
Workflow of the process underlying the creation of the CANT-HYD HMM Database.

As some group sizes were in the order of 10^5^ sequences and the computational requirement for aligning sequences grows exponentially with every added sequence, a nested clustering and phylogenetic pruning approach was implemented to overcome computational challenges for groups >5,000 sequences. The homologous group was first clustered at a lower identity (e.g., 50%) to reduce the size of the group, followed by alignment, phylogenetic reconstruction, and phylogenetic neighborhood pruning as described above. This process reduced the sequence search space around the archetype sequences by pruning the phylogenetic trees at a higher identity threshold. Because each sequence in a clustered group represents a group of sequences, for the selected pruned neighborhood, the clustered sequences were placed back in. Then the process of pruning the phylogenetic neighborhood of the reference sequence(s) was iterated using a higher clustering identity (e.g., 70%, 90%, etc.) until the prune group was ≤5,000 sequences or the clustering identity was raised to 98% (Figure 2), at which point the seed sequences for HMM creation were selected as described above.

### HMM Creation and Determination of Cutoff Scores

Because HMMs are sensitive to the alignment and position of each amino acid, the seed sequences that are now orders of magnitude smaller than the homologous groups they were derived from were realigned using a sensitive aligner, Clustal-Omega v1.2.4 (Sievers et al., 2011) followed by manual inspection of the alignment using Jalview v2.11.1.4 (Waterhouse et al., 2009), and generation of HMMs using the *hmmbuild* command of HMMER v3.2.1 (Eddy, 2011). The HMMs were used to search the archetype reference sequences and ‘DIAMOND Sequences’ database (Figure 2) using *hmmsearch* of HMMER v3.2.1 (Eddy, 2011). The domain scores of the hits to each HMM were plotted to visualize the frequency distribution pattern of the scores (Supplementary Figure 2). A “trusted” and a “noise” cutoff was chosen for each HMM using these score distributions. The trusted cutoff is the domain score above which a sequence can be confidently annotated for the function, as all experimentally verified genes used for the HMM scored above this cutoff. The noise cutoff was chosen to exclude genes that were predicted to have a different function. Thus, any hits scoring below the noise cutoffs are expected to *not* carry out the function represented by the HMM. Supplementary Table 2 includes information on genes that are the closest phylogenetic relatives to the archetype sequences of CANT-HYD HMMs.

### Validation of CANT-HYD HMMs Using Genomes of Known Hydrocarbon Degraders

Seventy-two genomes of microorganisms with published experimental evidence of an ability to degrade hydrocarbons were downloaded from GenBank and RefSeq (O’Leary et al., 2016) and categorized by the type of substrate and respiration (Supplementary Table 3). If the exact strain was not available, its closest relative from the Genome Taxonomy Database (GTDB) was chosen. For example, *Aromatoleum aromaticum* EbN1 anaerobically degrades aromatic compounds (Wöhlbrand et al., 2007). The genomes were searched using CANT-HYD HMMs and the resulting gene annotations, scoring above the trusted cutoff, were compared to the established degradation capability of the organism. If a gene hit multiple HMMs above the confidence threshold, it was assigned to the highest scoring HMM.

### Analysis of GTDB Genomes to Identify Potentially Novel Hydrocarbon Degrading Bacteria

The GTDB database (05-RS95 17th July 2020) (Parks et al., 2020) of representative bacterial and archaeal genomes was downloaded and searched using the CANT-HYD HMMs. For further investigation, gene sequences from cyanobacterial genomes with hits to LadA beta (above the noise cutoff) were combined with archetype reference sequences of long-chain alkane monooxygenases (LadA-alpha, LadA-beta, and LadB). The combined sequences were then clustered at 70% amino acid identity using USEARCH v9.0.2132_i86linux64 (Edgar, 2010) *cluster_fast.* Representative sequences of each cluster were then aligned using Muscle v3.8.31 (Edgar, 2004), followed by a maximum-likelihood phylogenetic reconstruction using FastTreeMP v2.1 (Price et al., 2010) (Supplementary Data Sheet 1).

### Analysis of Diverse Metagenomes Using CANT-HYD

Metagenomes representing diverse environments such as petroleum reservoirs (Hu et al., 2016; Nie et al., 2016; Liu et al., 2018; Christman et al., 2020), oil spill experimental microcosms (Tan et al., 2015; Dombrowski et al., 2016), marine systems (Orellana et al., 2017; Tully et al., 2018; Dong et al., 2019, 2020), host-associated microbiomes (Feigelman et al., 2017; Herman et al., 2020; Avila-Magaña et al., 2021), and other environments (Yao et al., 2017; Zorz et al., 2019), were downloaded either as unassembled raw data from the NCBI SRA or as predicted gene sequences from the JGI Genome Portal (Supplementary Table 4). Raw reads from unassembled metagenomes were filtered using BBDuk^1^ for a minimum quality of 15 and a minimum read length of 150 bp. Reads passing quality control were assembled using MEGAHIT (Li et al., 2015) with default parameters, followed by gene calling by Prodigal v2.6.3 (Hyatt et al., 2010) with the metagenomic option (*-p meta*). The amino acid sequences of predicted genes were searched against the CANT-HYD database using the *hmmsearch* command of HMMER v3.2.1 (Eddy, 2011) and only hits scoring above the noise cutoff for each HMM were visualized. Hit count for each metagenome was normalized by the total number of predicted genes.

### Comparison of CANT-HYD HMMs to Existing HMM Databases

The CANT-HYD HMMs were compared to equivalent HMMs from Pfam (Bateman et al., 2000), eggNOG (Huerta-Cepas et al., 2019) and KO (Kanehisa et al., 2016). Archetype sequences used to build CANT-HYD HMMs were annotated using eggNOG mapper (Cantalapiedra et al., 2021) with default parameters to identify and retrieve the closest eggNOG, Pfam and Kofam HMMs (Supplementary Table 5). These equivalent HMMs were used to annotate the isolate genomes, and hydrocarbon enrichment and host-associated metagenomes using *hmmsearch* (Eddy, 2011). Because suggested cutoffs were not included with eggNOG, Pfam, or KO database, an e-value cutoff of 10^–50^ was used to filter results.

## Results and Discussion

### Validation of CANT-HYD HMMs

**FIGURE 3:**
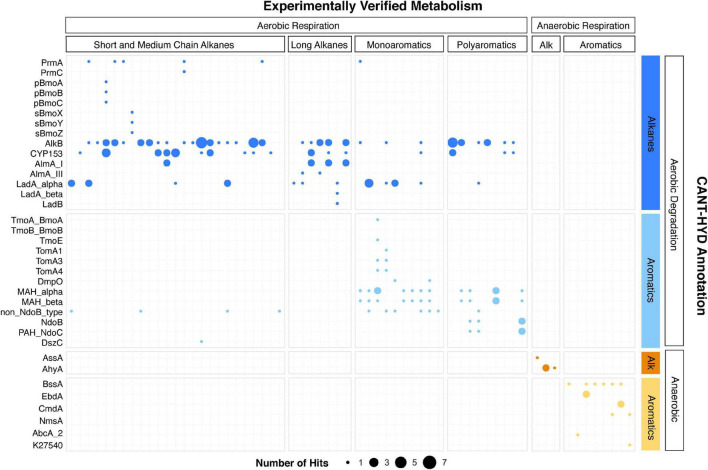
Concordance of CANT-HYD annotations of genomes and their experimentally verified hydrocarbon degradation activity. Each bubble represents hits above the confidence threshold from experientially verified hydrocarbon degrading genomes (x-axis) plotted against the HMMs of the CANT-HYD database (y-axis). The size of the bubble represents number of unique hits in the genome. The genomes (x-axis) and the CANT-HYD HMMs (y-axis) are organized by the hydrocarbon substrate and respiration. A complete list of isolate genomes, their Genbank accession, and their published hydrocarbon degradation capability can be found in Supplementary Table 3.

Genomes of 72 microorganisms with experimental evidence of hydrocarbon degradation were analyzed with the CANT-HYD HMMs for validation. For 62 out of 72 organisms, gene predictions using CANT-HYD were consistent with experimental data (Figure 3). Of the remaining 10 genomes, two genomes had hits with a score between the noise and trusted cutoffs, and eight genomes lacked hits above the noise cutoff. In a few instances, genomes were not available for the exact strain and a GTDB representative genome was used in their place. Although GTDB representatives share 95% average nucleotide identity with the cluster they represent, hydrocarbon degradation genes may be missing or different. Genomes of three organisms isolated on phenanthrene, chlorophenol and benzoate did not yield hits to any CANT-HYD HMMs. Although these substrates can be degraded by dioxygenases which share homology with mono- and polyaromatic ring hydroxylating dioxygenases, the lack of hits, even below the noise cutoff, indicates that the three organisms potentially use alternative metabolic pathways which were not covered by CANT-HYD. CANT-HYD predicted additional or unreported hydrocarbon substrate degradation capabilities for 16 genomes. For example, genes for toluene-2-monooxygenase (Tom) and toluene-4-monooxygenase (Tmo), and monoaromatic dioxygenase (MAH_alpha and MAH_beta) were found in the genome of *Pseudoxanthomonas spadix* BD-a59, a well-known benzene, toluene, ethylbenzene, and xylene (BTEX) degrader (Chun et al., 2010). Anaerobic hydrocarbon degradation genes were only detected in the genomes of anaerobes, further showing the prediction accuracy of CANT-HYD HMMs. Every HMM had at least one hit, except for the bacterial benzene carboxylase (AbcA_1) and toluene-benzene monooxygenase beta subunit (TmoB_BmoB). Anaerobic benzene degradation *via* benzene carboxylase (AbcA_1) has been identified in a single uncultured organism belonging to *Clostridia*, for which a genome is currently unavailable (Abu Laban et al., 2010). Toluene-benzene monooxygenase beta subunit (TmoB_BmoB) was found adjacent to TmoA_BmoA gene on the *Pseudoxanthomonas spadix* BD-a59 genome with a score above the noise cutoff, which indicates that it likely is a TmoB_BmoB gene divergent from the seed sequences that were used to make the HMM. Overall, these results show that CANT-HYD reliably identifies hydrocarbon degradation marker genes and can thus be used to predict the hydrocarbon degradation potential of genomes and in metagenomes.

### Diversity of Hydrocarbon Degrading Bacteria and Archaea

**FIGURE 4:**
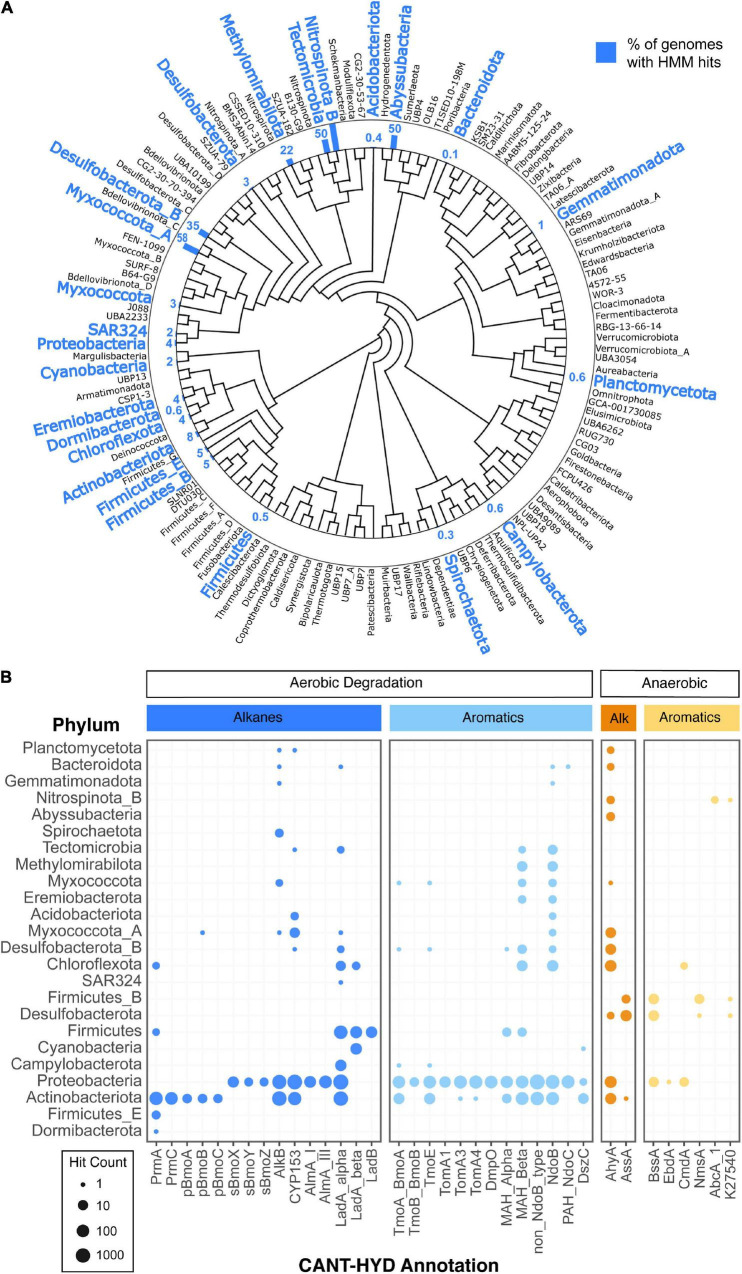
Diversity of hydrocarbon-degrading bacteria and archaea. GTDB representative genomes with CANT-HYD HMM hits shown on panel **(A)** the GTDB phylogenomic tree collapsed at phylum level. Phyla with genomes containing genes with a CANT-HYD HMM score greater than the noise cutoff are shown in blue. The corresponding bar and number indicates the percentage of the representative genomes in that phylum containing at least one hit to a CANT-HYD HMM. For example, Abyssubacteria contained two genome representatives in GTDB, and one of the genomes contained a high confidence match to a CANT-HYD HMM; thus, the bar shows 50%. **(B)** Distribution and number of hits for each phylum in GTDB across the CANT-HYD HMMs.

A large number of bacterial (30,238) and archaeal (1,672) genomes from the Genome Taxonomy Database (GTDB) were searched against the CANT-HYD HMMs (Parks et al., 2020). In total, 4,601 representative genomes from 18 bacterial phyla, had at least one hit to an HMM that scored higher than the trusted cutoff (Supplementary Table 2), and in total, 5,845 genomes from 24 bacterial phyla had hits to at least one CANT-HYD HMM above the noise cutoff (Figure 4). HMM hits from diverse bacterial phyla demonstrate the widespread potential for hydrocarbon degradation across bacteria (Figures 4A,B).

Many of these phyla contain no cultured representatives, and therefore annotation tools like CANT-HYD become important for offering clues about their metabolic potential. For instance, the potential for hydrocarbon degradation was found in genomes of poorly represented phyla including Abyssubacteria, Tectomicrobia, and Eremiobacterota (Figure 4B and Supplementary Figure 4). The phylum Abyssubacteria, often found in association with subsurface and hydrocarbon contaminated environments (Momper et al., 2018), had hits to anaerobic alkane degradation (AhyA). Eremiobacterota (formerly WPS-2), previously found in hydrocarbon enrichment cultures (Ramadass et al., 2018), had three members with aerobic aromatic hydrocarbon degradation potential (NdoB, MAH_alpha, MAH_beta). Two genomes from Tectomicrobia had high HMM scores to enzymes responsible for the aerobic degradation of monoaromatics (MAH_Beta), polyaromatics (NdoB), and long-chain alkanes (LadA_alpha and CYP153). There is currently no literature associating Tectomicrobia with hydrocarbon containing environments, however, high confidence matches to CANT-HYD HMMs suggest that they may have a previously unidentified role in the aerobic metabolism of a range of hydrocarbons.

#### Hydrocarbon Degradation in Archaea

Archaea contained fewer hydrocarbon degradation genes compared to bacteria. Only four genomes, all from the phylum Halobacteriota, had HMM hits above the trusted cutoff. Another 102 genomes, also from Halobacteriota, had at least one HMM hit above the noise cutoff (Supplementary Figure 3). The phylum Halobacteriota (formerly a member of phylum Euryarchaeota) is known to contain halophilic hydrocarbon degrading species (Al-Mailem et al., 2010; Oren, 2019). The identification of only a few archaeal hydrocarbon degraders may be due to either a lower representation of sequenced archaeal genomes, or an increased phylogenetic distance of archaeal hydrocarbon degradation genes to the primarily bacterial sequences that have been experimentally validated. Additionally, methanotrophy, the most well studied archaeal hydrocarbon degradation, is not covered by CANT-HYD. As more experimental evidence of archaeal genes emerge, the annotation of archaeal hydrocarbon degradation will improve.

#### Cyanobacteria as Alkane Degraders

**FIGURE 5:**
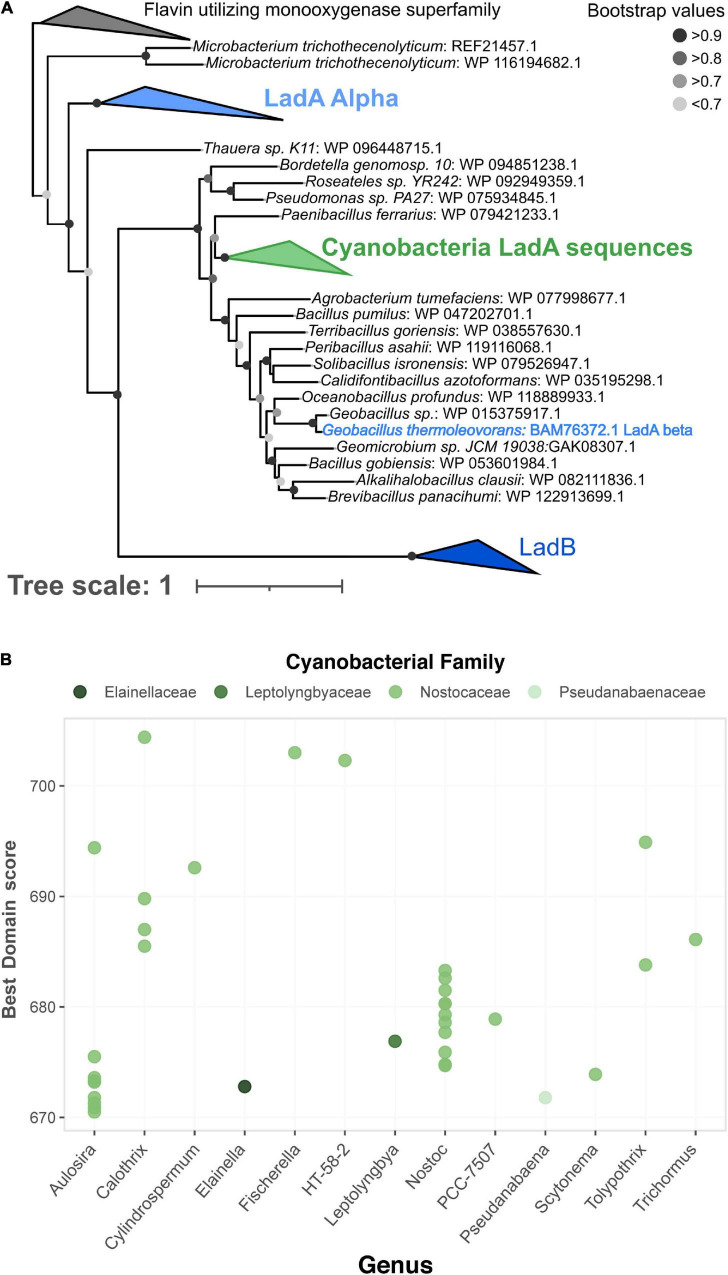
Detection of long-chain alkane monooxygenase (Lad) family of genes in Cyanobacteria. **(A)** Phylogenetic tree of Lad sequences including a cluster of 102 sequences detected in Cyanobacteria genomes by CANT-HYD (green). **(B)** Distribution of scores for LadA beta HMM hits in Cyanobacteria genomes. Original tree file for is available as Supplementary Data Sheet 1.

Thirty-six genes from 29 cyanobacterial GTDB representative genomes, mostly from the family Nostocaceae and the genera *Nostoc* and *Aulosira*, were predicted to contain LadA beta, a long-chain alkane monooxygenase (Figure 5). LadA beta is one of the three LadA-type long-chain alkane monooxygenase enzymes with experimental evidence of long-chain alkane degradation (Boonmak et al., 2014). Phylogenetically, these cyanobacterial genes were related to the experimentally verified LadA beta sequence from *Geobacillus thermoleovorans* (BAM76372.1), suggesting that the genes perform a similar role in their photosynthetic hosts (Figure 5A). Many cyanobacterial species produce long-chain alkanes, potentially at globally relevant levels (Lea-Smith et al., 2015; Love et al., 2021), and alkane degradation has been observed in microbial communities with abundant cyanobacteria (Abed, 2010). Thus far however, it has been inconclusive whether the alkane degradation is performed by cyanobacteria or other heterotrophic community members, and if the cyanobacteria are responsible, which degradation pathways they utilize (Al-Hasan et al., 1998; Qiao et al., 2020). Strong hits to LadA beta suggest that some cyanobacterial species have the metabolic potential for long-chain alkane degradation *via* LadA, although experiments are needed to confirm if this genetic potential is realized.

### Hydrocarbon Degradation in Diverse Environments

**FIGURE 6:**
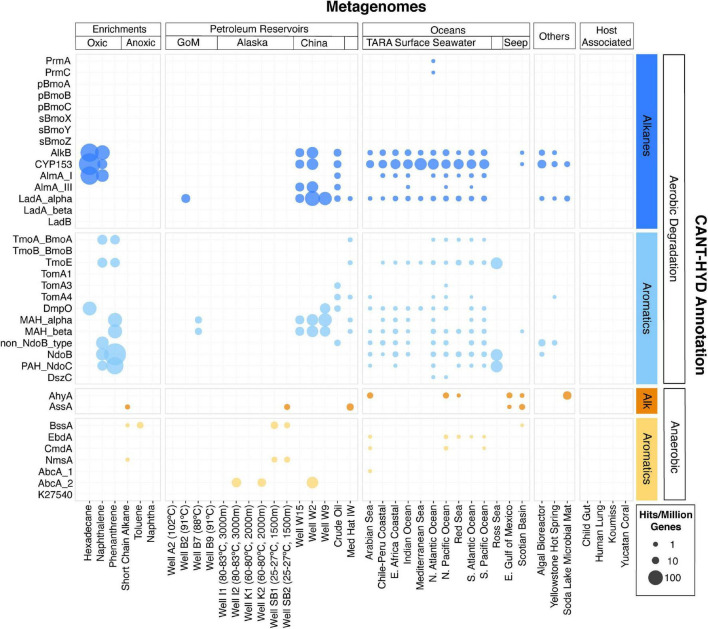
Metagenomes from diverse environments analyzed with the CANT-HYD HMMs. CANT-HYD HMM hits (scores ≥ noise cutoff) (y-axis) to each metagenome (x-axis) normalized to per million protein coding genes. The size of the bubble depicts proportion of hydrocarbon degrading genes to total genic content of the metagenome. The metagenomes (x-axis) are grouped under Enrichments (hydrocarbon-degrading enrichment cultures), Petroleum Reservoirs (produced well water from petroleum reservoirs from Alaska, Gulf of Mexico, Jiangsu and Qinghai, China, and Medicine Hat, Alberta), Oceans (cold seeps, TARA surface seawater), Host-Assoc (host-associated microbiomes), Other: (other environments). The CAN-HYD HMMs are grouped by hydrocarbon substrate (alkane and aromatic) and respiration (aerobic and anaerobic). See Supplementary Table 4 for details regarding these metagenomes.

The CANT-HYD HMMs were used to search for hydrocarbon degradation potential in 41 metagenomes, representing diverse environments including hydrocarbon degrading enrichment cultures, petroleum reservoirs, oceans, host-associated microbiomes, alkaline lakes, and hot springs. Hydrocarbon degradation genes were detected in all these environments, except for the host-associated microbiomes, which are presumed to have a limited presence of hydrocarbons (Figure 6).

When normalized for total gene content, the highest proportion of hydrocarbon degradation genes were detected in the metagenomes of hydrocarbon-degrading enrichments. Genes for aerobic alkane degradation, such as AlkB, CYP153, and AlmA, were the most widely detected in this dataset. Many marker genes for aerobic hydrocarbon degradation were found ubiquitously in metagenomes from ocean surface waters, while genes for anaerobic hydrocarbon degradation were largely detected in metagenomes sequenced from anoxic habitats such as petroleum reservoirs, subseafloor sediments, and anoxic hydrocarbon degrading microcosms. Further, some degradation enzymes covered by the CANT-HYD HMMs yielded no hits, namely butane monooxygenases (pBmoA, pBmoB, and pBmoC, and sBmoX, sBmoY, and sBmoZ), and benzene and naphthalene carboxylases (AbcA_1 and K27540). These HMMs were made with less than five seed sequences as they had only a few close relatives (≥50% sequence identity) in the public nucleotide database at the time of this work.

#### Enrichment Cultures of Hydrocarbon Degrading Microorganisms

Genes from the glycyl radical enzyme family (Ass/Bss/Nms) for anaerobic hydrocarbon degradation were identified in two out of the three anoxic cultures, namely the toluene and short-chain-alkane enrichments (Tan et al., 2015; Figure 6). The number of genes identified in this study were half of those reported in the original, which were also annotated using custom HMMs (Table 1). Observed discrepancies include the detection of partial gene sequences in the metagenomic assemblies (Table 1: denoted with an *) (Tan et al., 2015). Partial gene sequence matches to the CANT-HYD Ass/Bss/Nms HMMs scored below the noise cutoffs and did not pass the threshold. Partial hits to HMMs scoring below the noise cutoffs cannot be reliably annotated as they may be a partial sequence of a related gene that encodes an enzyme with a different function. In this case, the partial hits for genes encoding for glycyl radical enzymes (Ass/Bss/Nms) can be functionally similar (Supplementary Figure 1) to and share high sequence homology with pyruvate formate lyase. While CANT-HYD will not perform optimally with unassembled, partially or poorly assembled data, the noise and trusted cutoffs are recommended for a low false-positive rate, by filtering out partial genes that risk being misannotated.

**TABLE 1:** Number of AssA, BssA, and NmsA genes detected in this analysis compared to the original study (Tan et al., 2015).

Substrate	Short-chain alkanes		Toluene		Naphtha	
	
Genes	Original study	CANT-HYD	Original study	CANT-HYD	Original study	CANT-HYD
**AssA**	4 + 1*	4	1*	–	1*	–
**BssA**	3	1	1	1	1 + 2*	–
**NmsA**	1	1	–	–	1*	–

Asterisk (*) indicates partial gene homologs.

Several genes for aerobic hydrocarbon degradation were identified in oxic cultures inoculated with the Deepwater Horizon oil plume and enriched on hexadecane, naphthalene, and phenanthrene as hydrocarbon substrates (Figure 6). Genes for the aerobic degradation of medium and long-chain alkanes (CYP153, AlkB, and AlmA_GroupI) were detected in the hexadecane and naphthalene enrichments, while a variety of genes for aromatic hydrocarbon degradation were detected in the oxic naphthalene and phenanthrene enrichment cultures.

#### Petroleum Reservoirs and Hydrocarbon Biodegradation

Multiple marker genes were identified in all metagenomes from petroleum reservoirs, except for four reservoirs that were either at a high temperature (>80°C) or were deep subsurface (Figure 6). Produced water metagenomes from petroleum reservoirs in Alaska (wells SB1, SB2, K1, K2, I1, and I2) were found to contain only anaerobic hydrocarbon degradation genes in agreement with the associated study (Hu et al., 2016). CANT-HYD further detected the presence of a putative benzene carboxylase gene (AbcA_2) in the metagenomes from two of the oil wells (K2 and I2), which was not reported in the original study. These oil reservoirs were reported to contain complete and partial genomes of a sulfide-producing archaeon, *Archaeoglobus*, and our results indicate that it can potentially metabolize benzene anaerobically.

Similar observation of an *Archaeoglobus* metagenome-assembled-genome (MAG) and its association with a benzene carboxylase (AbcA_2) gene comes from the metagenome of well W2 from the Jiangsu Oil Reservoir, China (Liu et al., 2018). Although the original study found alkyl succinate synthase genes (Ass) in an *Archaeoglobus* MAG, in this study, genes for the glycyl radical family of enzymes from these metagenomes and the *Archaeoblogus* genome scored below the noise cutoff. Any hits below the noise cutoff cannot be reliably annotated automatically and therefore require manual curation, such as using gene phylogeny to differentiate between glycyl radical enzyme and pyruvate formate lyase. A study by Khelifi et al. (2014) also shows evidence for anaerobic long-chain alkane degradation by *Archaeoglobus*, however, the genes identified as responsible for this metabolism share low sequence homology with bacterial alkyl succinate synthase alpha subunit and will not be annotated by the CANT-HYD AssA HMM. Therefore, a separate HMM for archaeal alkyl succinate synthase alpha subunit would be required when strong experimental evidence for these genes becomes available. Several genes for aerobic alkane and monoaromatic hydrocarbon degradation (Figure 6) were also identified in the Well W2 metagenome, which were not originally reported. These findings highlight the utility of CANT-HYD, which can search for a comprehensive suite of hydrocarbon degradation markers, independent of *a priori* knowledge of the system.

#### Widespread Hydrocarbon Degradation Potential in Global Surface Seawaters

Widespread potential for aerobic hydrocarbon degradation was detected in the surface seawater metagenomes collected by the TARA Oceans survey (Karsenti et al., 2011; Tully et al., 2018) and other studies (Orellana et al., 2017). Predicted hydrocarbon metabolism was largely driven by medium- and long-chain alkane hydroxylases, and ring-hydroxylating dioxygenases. This pervasive metabolic capability in the global ocean surface could be the result of biogenic alkanes synthesized by cyanobacteria (Lea-Smith et al., 2015) or the accumulation of polyaromatic hydrocarbons by other ocean phytoplankton, resulting in a “cryptic hydrocarbon cycle” (Binark et al., 2000; Love et al., 2021). An ocean metagenome from the Ross Sea, Antarctica had an exceptionally high abundance of marker genes involved in polyaromatic hydrocarbon degradation. The Ross Sea is well-known for its seasonal algal blooms and rapid carbon turnover (Smith et al., 2000; Peloquin and Smith, 2007), which have been associated with biogenic alkanes, polyaromatic hydrocarbons, and PAH degraders (Gutierrez et al., 2011; Lea-Smith et al., 2015; Love et al., 2021). Overall, our findings support the recent experimental evidence of a marine hydrocarbon cycle (Love et al., 2021).

#### Comparison of CANT-HYD HMMs to Existing HMM Databases

The annotation performance of CANT-HYD HMMs was compared to HMMs from Pfam, KO, and eggNOG databases. As the purpose-built CANT-HYD database is curated for hydrocarbon degradation genes, it assigned more specific annotations than Pfam, eggNOG and KO databases. Pfam and eggNOG often annotated genes as the broad protein family to which the hydrocarbon degradation genes belong. Examples of these generic descriptions included “monooxygenase” for long-chain alkane monooxygenase (LadA), and “pyruvate-formate-lyase like protein” for the AssA, BssA, and NmsA genes (Supplementary Table 5). While these descriptions are broadly accurate, they do not give sufficient information about the gene for strong functional inference. The KO database assigned more specific annotations of the enzyme function compared to eggNOG and Pfam, but not necessarily to the same level of substrate specificity as the CANT-HYD HMMs. The KO database also missed annotations to more recently discovered hydrocarbon degradation genes such as naphthalene carboxylase (K27540), and long-chain alkane monooxygenase beta (LadA beta) (Supplementary Table 5).

**FIGURE 7:**
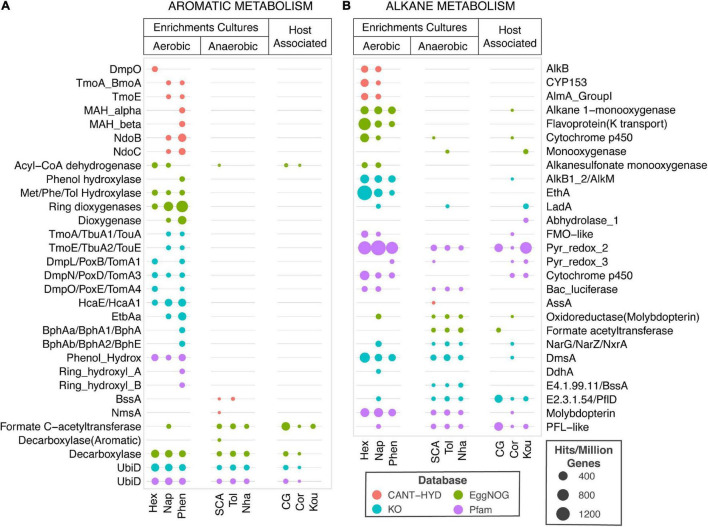
Comparison of hydrocarbon metabolism prediction in metagenomes. Hits to aromatic hydrocarbon **(A)** and alkane **(B)** metabolism HMMs (y-axis) from CANT-HYD (red), eggNOG (green), KO (teal), and Pfam (purple) to each metagenome (x-axis) normalized to per million coding genes. The metagenomes (x-axis) are grouped under Enrichment Cultures or Host associated microbiome. The enrichment culture metagenomes representing microbial community grown on under aerobic conditions on hexadecane (Hex), naphthalene (Nap), or phenanthrene (Phen) or under anaerobic conditions on short-chain alkanes (SCA), Toluene (Tol), or Naphtha (Nha). The host-associated microbiomes are from child gut (CG), Coral (Cor), or Koumiss (Kou). The size of the bubble depicts proportion of hydrocarbon degrading genes to total genic content of the metagenome. See Supplementary Table 5 for details regarding the HMMs and the underlying data.

The equivalent HMMs from the three databases were compared with CANT-HYD HMMs using genomes of 62 experimentally verified hydrocarbon degrading isolates and 10 metagenomes from hydrocarbon enrichments and host associated microbiomes. This search resulted in the 1000s of hits to from the Pfam, eggNOG and KO database and hence to remove spurious matches and increase confidence in annotations, only hits with an e-value below 10^–50^ were retained for analysis (Figure 7 and Supplementary Table 5). After filtering, Pfam, eggNOG and KO still reported >1,000 hits for 62 genomes, while the total number of genes identified by CANT-HYD above the trusted cutoff was only 190. Furthermore, as previously mentioned that not all HMMs of expected specific metabolisms were found in these public databases, a large proportion of hits were to HMMs describing broad protein families such as “cytochrome p450,” “Pyr_redox_2,” and “monooxygenase.” A search of the metagenomes resulted in a total of 925, 525, and 463 genes identified by Pfam, KO, and eggNOG HMMs, respectively. In contrast, only 50 genes were identified in the same dataset using the noise cutoff of the CANT-HYD HMMs. These discrepancies in identified genes in genomes and metagenomes originates from the differences in the HMM targets of the databases. The lack of specificity of Pfam, eggNOG, and some KO is likely to detect genes that are related to hydrocarbon degradation genes, but not involved in hydrocarbon degradation. For the same reasons, while all other databases produced hits in host-associated metagenomes, CANT-HYD did not (Figure 7). Together, these comparisons highlight the usefulness and accuracy of CANT-HYD to identify and annotate specific hydrocarbon metabolic potential by using curated cutoffs for HMMs designed specifically for hydrocarbon degradation marker genes.

## Conclusion

Here, we describe CANT-HYD, an HMM database of marker genes for hydrocarbon degradation. These phylogenetically informed HMMs accurately identify over 37 genes relevant to aerobic and anaerobic metabolisms of aliphatic and aromatic hydrocarbons in genomes and metagenomes. Each CANT-HYD HMM includes a manually curated trusted and noise cutoff score for automated reliable detection of these hydrocarbon degradation marker genes. To the best of our knowledge, CANT-HYD is the first dedicated tool for annotation of hydrocarbon degradation genes in genomes and metagenomes. We demonstrate the use of CANT-HYD as an exploratory tool by surveying all genomes in GTDB (30,238 bacterial and 1,672 archaeal), as well as several large metagenomic datasets. We uncovered the potential for long-chain alkane degradation in some cyanobacterial genomes and identified widespread potential for aerobic hydrocarbon degradation in global ocean surface waters, supporting a recently discovered marine hydrocarbon cycle. The comparison to other publicly available HMMs highlights the need for a curated HMM database for specific and precise annotation of hydrocarbon degradation genes and large-scale detection of hydrocarbon degrading capabilities in genomes and metagenomes.

## Data Availability Statement

The original contributions presented in the study are included in the article/Supplementary Material, further inquiries can be directed to the corresponding author.

## Author Contributions

VK, JZ, DAG, AC, EB, MAB, AJP, AKH, BN, and SB carried out the literature review and sequence data searching. VK, JZ, DAG, AC, EB, MAB, AJP, and SB made and validated the HMMs. VK, JZ, DAG, AC, EB, and SB wrote the manuscript. VK, JZ, DAG, AC, MAB, and SB performed the genomic and metagenomic data analyses. VK, JZ, AC, and SB made the figures. BN and MS conceived the study. DAG created and maintained the GitHub archive. All authors contributed toward methodology development and editing and reviewing the manuscript.

## Conflict of Interest

The authors declare that the research was conducted in the absence of any commercial or financial relationships that could be construed as a potential conflict of interest.

## Publisher’s Note

All claims expressed in this article are solely those of the authors and do not necessarily represent those of their affiliated organizations, or those of the publisher, the editors and the reviewers. Any product that may be evaluated in this article, or claim that may be made by its manufacturer, is not guaranteed or endorsed by the publisher.

## Footnotes

1 Bushnell, B. *BBTools*. Available online at: https://jgi.doe.gov/data-andtools/bbtools/

## Abbreviations

CANT-HYD: Calgary approach to ANnoTating HYDrocarbon degradation genes
HMM: Hidden Markov Models
GTDB: Genome Taxonomy Database
BTEX: Benzene, Toluene, Ethyl Benzene, and Xylene.
