# Supplementary material for: CANT-HYD: A Curated Database of Phylogeny-Derived Hidden Markov Models for Annotation of Marker Genes Involved in Hydrocarbon Degradation
Source: Front Microbiol. 2022 Jan 7;12:764058. doi: 10.3389/fmicb.2021.764058 (PMC8767102; doi:10.3389/fmicb.2021.764058)
Supplement: Supplementary file 2 [file Data_Sheet_2.docx]

**SUPPLEMENTARY FIGURES**


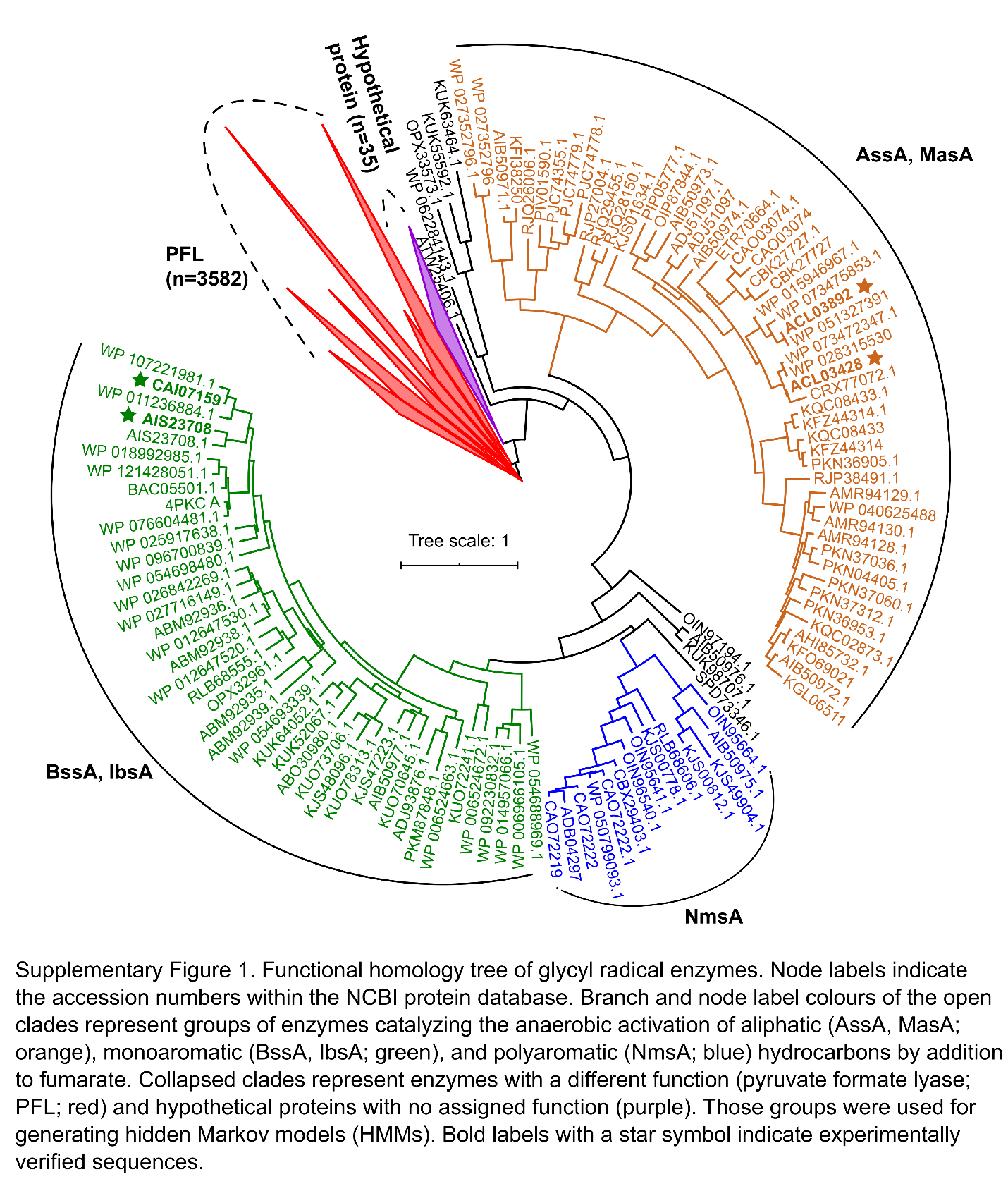


**Supplementary Figure S1. Functional hormonology of glycyl radical family of genes**. Branch and node label colors indicate sequences belonging to AssA (Orange), BssA/IbsA (green), and NmsA (blue) genes that catalyze anerobic degradation of aliphatic, monoaromatic, and polyaromatic hydrocarbons, respectively. Collapsed nodes represent phylogenetically related, functionally different genes belonging to pyruvate formate lyase (Pfl; red) and hypothetical proteins (purple). Bold labels with a star symbol indicate experimentally verified sequences. Node labels indicate NCBI accession number.


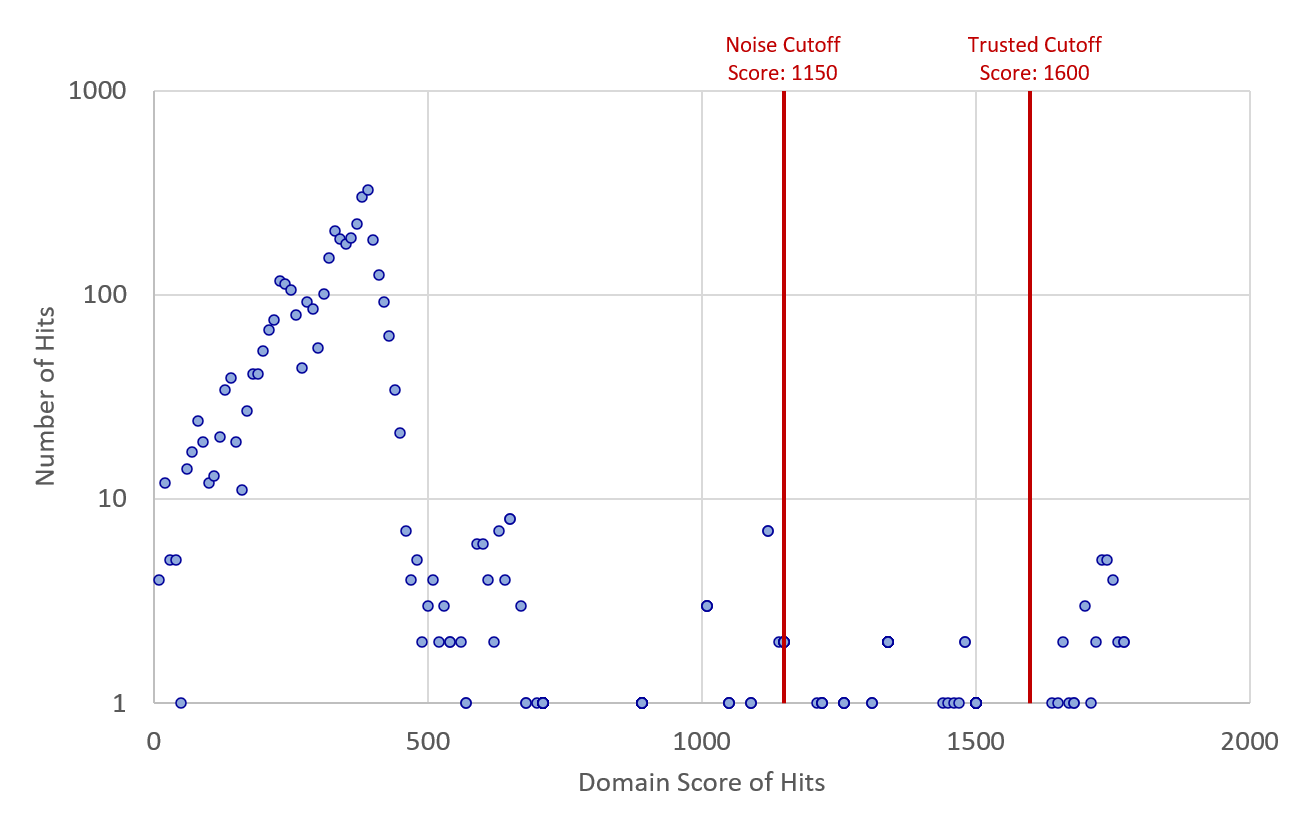


**Supplementary Figure S2. HMM score distribution of NCBI subset run against the CANT-HYD BssA HMM**. This is an example of how the trusted and noise cutoff score was chosen for all CANT-HYD HMMs. The frequency of hit domain scores was plotted against domain scores. The trusted cutoff score was selected after the peak (for BssA: 1600) that included all the experimentally verified sequences. The noise cutoff was selected before the peak (For BssA: 1150) that contains functionally different sequences. Thus, the cutoffs ensure that all the experimentally verified archetype sequences were included in the trusted cutoff and the noise cutoff excludes spurious hits.


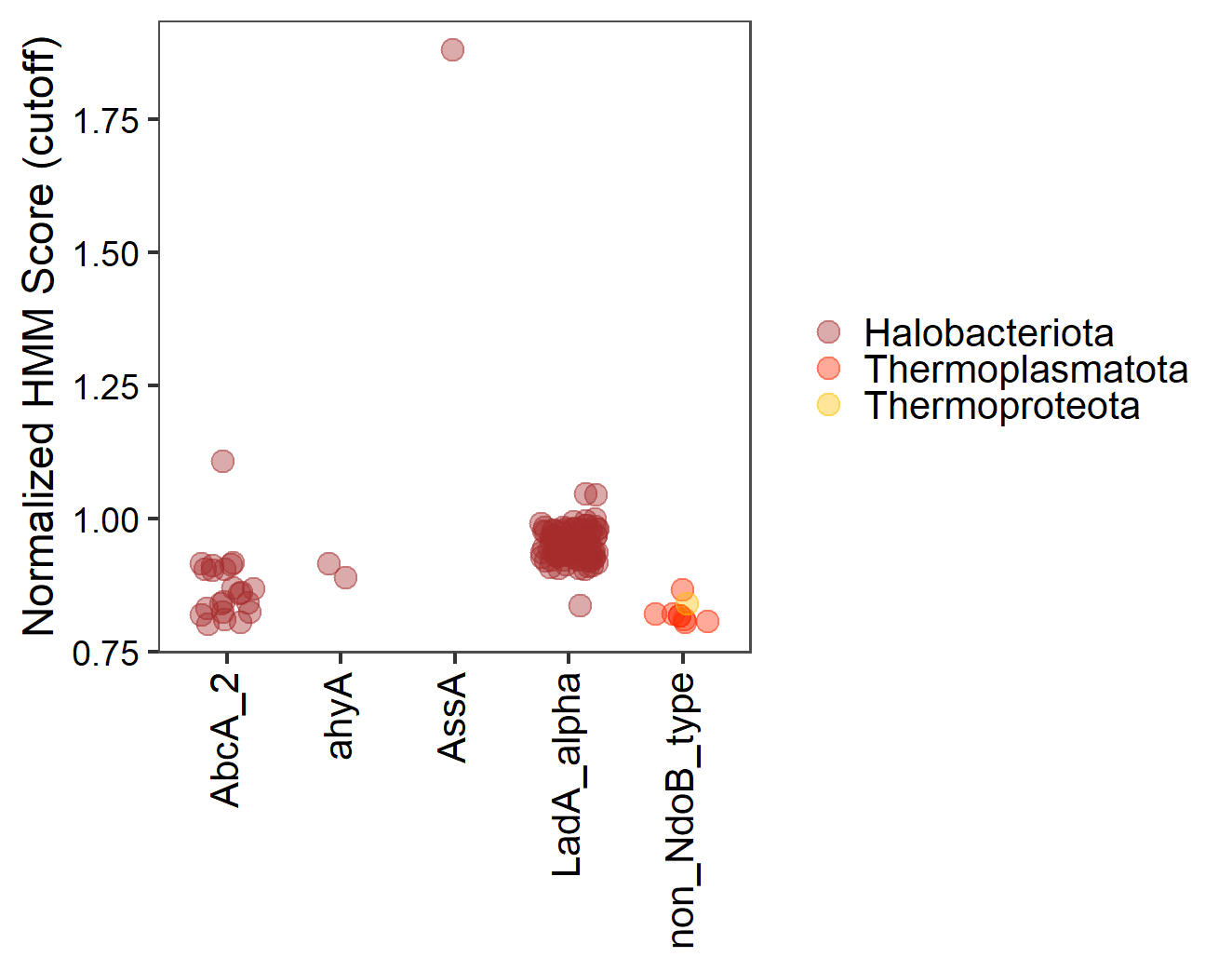


**Supplementary Figure S3. Distribution of CANT-HYD HMM scores among archaeal representative genomes in GTDB**. Only HMM hits with the best domain score above 80% of the noise cutoff were plotted.


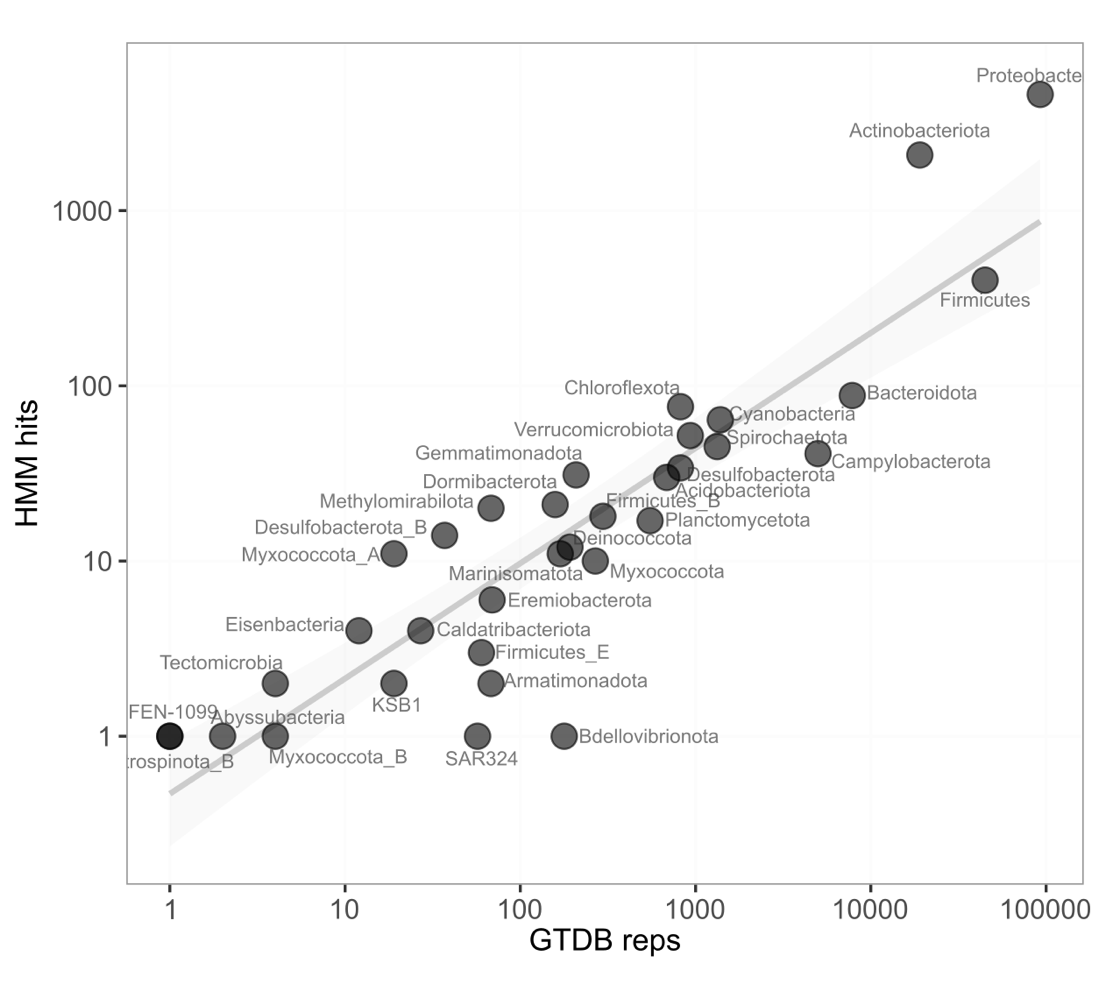


**Supplementary Figure S4**. Distribution of phyla with high-confidence hits (> 80% noise cutoff) plotted against the number of CANT-HYD HMM hits and the number of GTDB representative species. Phyla above the line contain more species with hydrocarbon containing potential than the average of all phyla identified with hydrocarbon degrading potential.
