# Supplementary material for: CANT-HYD: A Curated Database of Phylogeny-Derived Hidden Markov Models for Annotation of Marker Genes Involved in Hydrocarbon Degradation
Source: Front Microbiol. 2022 Jan 7;12:764058. doi: 10.3389/fmicb.2021.764058 (PMC8767102; doi:10.3389/fmicb.2021.764058)
Supplement: Supplementary file 3 [file Data_Sheet_3.docx]

**Supplementary Information**

**CANT-HYD: A curated database of phylogeny-derived Hidden Markov Models for annotation of marker genes involved in hydrocarbon degradation**

Varada Khot^1†^, Jackie Zorz^1†^, Daniel A. Gittins^2^, Anirban Chakraborty^2^, Emma Bell^2^, María A. Bautista^2^, Alexandre J. Paquette^1^, Alyse K. Hawley^1^, Breda Novotnik^1^, Casey R. J. Hubert^2^, Marc Strous^1^, Srijak Bhatnagar^2*^

^1^ Department of Geoscience, University of Calgary, Calgary, Alberta, T2M 4G5, Canada

^2^ Department of Biological Sciences, University of Calgary, Calgary, Alberta, T2M 4G5, Canada

**DESCRIPTION OF CANT-HYD HMMS**

A literature search led to the identification of 105 genes involved in aerobic and anaerobic degradation of alkanes and aromatic hydrocarbons. With these genes as the starting point, the procedure of workflow in Fig. 1 resulted in the design of 37 Hidden Markov Models (HMMs), describing these 105 functional genes. Together, these HMMs make up the Calgary approach to ANnoTating HYDrocarbon degrading enzymes (CANT-HYD) database. Fifteen HMMs were associated with aerobic alkane degradation, 13 with aerobic aromatic hydrocarbon degradation 2 with anaerobic alkane degradation, and 7 with anaerobic aromatic hydrocarbon degradation (Figure 2).

*Aerobic degradation of alkanes*

The aerobic degradation of alkanes includes the oxygenation of methyl groups to the corresponding alcohol. Oxygenase enzymes catalyzing this reaction vary based on alkane structure and chain length [51], which are divided here in two broad groups: short and medium chain alkanes (≤ C_16_) and long chain alkanes (≥ C_17_) [52].

Monooxygenation of volatile alkanes (< C_5_) is performed by enzymes that are either copper-containing membrane proteins (particulate) [53] or soluble non-heme diiron monooxygenases [54,55]. Of these enzymes, methane monooxygenases have been extensively studied and are well-characterized [56,57] with good, existing HMMs [58], and so were not included in CANT-HYD. Ethane degradation has been identified in some methanotrophs [59,60] and it is speculated to involve copper-containing membrane monooxygenases (CuMMOs) that may have a broad substrate range [61]. However, specific enzymes for ethane degradation have not yet been characterized and therefore ethane degradation is not covered in CANT-HYD.

Degradation of propane is catalyzed by a two-subunit soluble diiron enzyme, propane 2-monooxygenase. Both the large subunit (PrmA) and the small subunit (PrmC) [62] constitute the catalytic center and an HMM was created for each. Two different enzymes, particulate and soluble butane monooxygenase, catalyze the conversion of butane to butanol. Particulate butane monooxygenase (pBMO) is a membrane protein with sequence similarity to particulate methane monooxygenase (pMMO) [53,63]. HMMs were made for each subunit of the pBMO trimer. The soluble butane monooxygenase (sBMO) is a three-component diiron monooxygenase, with sequence similarity to soluble methane monooxygenase (sMMO) [55]. The catalytic hydroxylase (BMOH) of this complex is made of up of three subunits, BmoX, BmoY, and BmoZ. An HMM was made for each of these subunits.

Degradation of C_5_–C_16_ alkanes can be catalyzed by AlkB and CYP153 [64]. AlkB is a membrane-bound non-heme diiron monooxygenase that catalyzes alkane hydroxylation using a rubredoxin and a rubredoxin reductase for electron transfer. The rubredoxin component can be fused to the hydroxylase subunit in some organisms [64], so the AlkB HMM was manually curated to exclude the rubredoxin component. A separate HMM was made for CYP153, a cytochrome P450 that catalyzes alkane hydroxylation using ferredoxin and ferredoxin reductase for electron transfer [64,65]. Similar to AlkB, the CYP153 HMM was created from a manually curated seed sequence alignment to exclude its ferredoxin and ferredoxin reductase components.

Oxidation of long chain alkanes (≥ C_17_) can be catalyzed by two groups of enzymes, LadA [66] or AlmA [67,68]. The LadA-type family of enzymes catalyzes oxidation of C_15_–C_36_ alkanes. In our phylogenetic reconstruction, the experimentally verified sequences of the catalytic subunits (LadA *alpha*, LadA *beta*, and LadB) shared little sequence homology, therefore, an HMM was created for each. AlmA, another long chain alkane oxidizing enzyme, has a substrate specificity of C_20_–C_32_ alkanes [67,68]. Based on the phylogeny, CANT-HYD includes two HMMs corresponding to AlmA group I and AlmA group III from the classification system proposed by Wang and Shao [68].

*Anaerobic degradation of alkanes*

The best understood pathway in the anaerobic degradation of hydrocarbons includes as its first step the addition to fumarate, catalyzed by a glycyl-radical enzyme (Figure 2B) [69][70]. Glycyl radical enzymes, common in anaerobic biochemical reactions, share sequence homology, despite acting on different substrates. The glycyl-radical enzyme that initiates anaerobic alkane oxidation is alkyl succinate synthase or Ass (also sometimes referred to as methyl-alkyl succinate synthase, Mas). CANT-HYD includes an HMM for the catalytic alpha subunit (AssA).

Recently, another anaerobic alkane degradation pathway has been proposed that uses hydroxylation and shares sequence homology with the anaerobic aromatic hydroxylase, ethylbenzene dehydrogenase (Ebd) [7]. The enzyme, alkane C2 methylene hydroxylase (Ahy), is postulated to catalyze the oxidation of a non-terminal carbon atom of the alkane. An HMM was made for the catalytic alpha subunit, AhyA, using a reference sequence with putative evidence of this degradation pathway [69] (Figure 2B).

*Aerobic degradation of aromatic hydrocarbons*

The pathways involved in the aerobic degradation of aromatic hydrocarbons can be divided into two main categories, hydroxylation or oxidation of the aromatic ring. CANT-HYD includes HMMs for marker genes that are involved in the hydroxylation of phenol (DmpO) and toluene/xylene (TomA1, TomA2, and TomA3) [55]. Oxidation of the aromatic ring triggers a degradation cascade leading to ring cleavage. Dioxygenases that catalyze first oxygen attack on a wide variety of aromatic hydrocarbons share an evolutionary ancestry [71]. Dioxygenase~~,~~ phylogeny revealed those that catalyze monoaromatic hydrocarbons (MAH) formed a distinct monophyletic clade distant from the monophyletic clade of polyaromatic hydrocarbon (PAH) dioxygenases. Within the monophyletic clades, there was no substrate specific subclade of dioxygenases. MAH and PAH dioxygenases can oxidize several substrates [71,72], therefore, HMMs built for aerobic aromatic degradation genes (MAH_alpha, MAH_beta, NdoB, and NdoC) are applicable to a group of substrates (Figure 2C). One additional HMM was created for a putative beta subunit of PAH dioxygenases (non-NdoB) that share little sequence homology to the NDO system [71].

One exception to the phylogenetic split of monoaromatic and polyaromatic dioxygenases were the biphenyl dioxygenases (bph). All experimentally verified sequences for the bph system were found to be within the monoaromatic clade, indicating a previously reported shared evolutionary history of biphenyl dioxygenases to toluene dioxygenases [71]. Furthermore, an HMM was made for dibenzothiophene metabolism (DszC), to include this well studied organosulfur petroleum compound [73].

*Anaerobic degradation of aromatic hydrocarbons*

The activation step for the anaerobic degradation of aromatic hydrocarbons can be carried out by three processes: fumarate addition, hydroxylation (addition of water), and carboxylation (addition of CO_2_). The catalytic subunits of fumarate-adding enzymes that act on aromatic substrates form two distinct clusters with corresponding substrate specificity: benzyl succinate synthase (BssA) and naphthyl succinate synthase (NmsA), which are phylogenetically related to alkyl succinate synthase (AssA, see anaerobic degradation of alkanes). CANT-HYD includes an HMM for each of these two groups (Figure 2D).

Anaerobic hydroxylation is the first step in the degradation of ethylbenzene (by ethylbenzene dehydrogenase, Ebd) and p-cymene (by p-cymene dehydrogenase, Cmd) [7,74] The subunit of experimentally verified dehydrogenases that facilitate this catalysis formed distinct phylogenetic clades based on substrate specificity. Thus, two separate HMMs were created, targeting the catalytic subunit of these dehydrogenases.

Lastly, it has been proposed that under anoxic conditions, aromatic hydrocarbon degradation can proceed with carboxylation as the initial activation step (Figure 2D) [75]. A putative benzene carboxylase (Abc) has been identified in both bacteria and archaea [76,77]. Similarly, a naphthalene carboxylase (K27540) has been proposed for the carboxylation of naphthalene. Three HMMs were created for the putative catalytic subunits of these carboxylases, two targeting benzene, AbcA_1 (bacterial) and AbcA_2 (archaeal), and a third for naphthalene carboxylase (K27540).

**Supplementary Tables and Data**

Table ST1. Information on the experimentally verified reference sequences used in creation of CANT-HYD HMMs. (Excel file)

Table ST2. List of CANT-HYD HMMs including corresponding cutoff scores and nearest related gene. (Excel file)

Table ST3. Information on genomes of know hydrocarbon degrader used in analyses. (Excel file)

Table ST4. List of metagenomes used in analyses. (Excel file)

Table ST5. Results of the HMM comparison analysis between CANT-HYD HMMs and the corresponding HMMs from Pfam, eggNOG, and KO databases. (Excel file)

Data SD1. Long-chain alkane monooxygenase (LAD) tree file including cyanobacterial genes and experimentally verified LAD sequences. (.tre file)
